# Scalability, test–retest reliability and validity of the Brief INSPIRE-O measure of personal recovery in psychiatric services

**DOI:** 10.3389/fpsyt.2024.1327020

**Published:** 2024-05-14

**Authors:** Stine Bjerrum Moeller, Pia Veldt Larsen, Stephen Austin, Mike Slade, Ida-Marie T. P. Arendt, Martin Stolpe Andersen, Sebastian Simonsen

**Affiliations:** ^1^ Psychotherapeutic Center Stolpegård, Region Hovedstad Psychiatry, København, Denmark; ^2^ Department of Psychology, Faculty of Health Sciences, University of Southern Denmark, Odense, Denmark; ^3^ Department of Trauma and Torture Survivors, Mental Health Services of Southern Denmark, Vejle, Denmark; ^4^ Mental Health Services in the Region of Southern Denmark, Vejle, Denmark; ^5^ Psychiatric Research Unit, Psychiatry Region Zealand, Slagelse, Denmark; ^6^ Institute of Mental Health, University of Nottingham, Nottingham, United Kingdom; ^7^ Faculty of Nursing and Health Sciences, Nord University, Bodø, Norway; ^8^ Danish Psychological Publishing, Copenhagen, Denmark; ^9^ Department of Psychology, Faculty of Social Sciences, University of Copenhagen, Copenhagen, Denmark

**Keywords:** mental health, psychometrics, INSPIRE, personal recovery, transdiagnostic, Mokken, CHIME, Patient Reported Outcome Measure (PROM)

## Abstract

**Introduction:**

Mental health services have transitioned from treating symptoms to emphasizing personal recovery. Despite its importance, integrating personal recovery into clinical practice remains work in progress. This study evaluates the psychometric qualities of the Brief INSPIRE-O, a five-item patient-reported outcome measure assessing personal recovery.

**Method:**

The study collected data from 2018 to 2020 at the Mental Health Services, Capital Region of Denmark, using an internet-based system examining 8,192 non-psychotic patients – receiving outpatient treatment.

**Materials:**

This study evaluated the Brief INSPIRE-O and used measures of symptomatology (SCL-10), well-being (WHO-5), and social functioning (modified SDS).

**Results:**

The study population comprised 76.8% females with a mean age of 32.9 years, and diagnoses included anxiety (28%), depression (34%), and personality disorder (19%). The mean Brief INSPIRE-O score (39.9) was lower than the general population norm (71.1). The Brief INSPIRE-O showed acceptable test–retest reliability (0.75), scalability (0.39), and internal consistency (0.73). Correlations with other mental health criteria were in the expected direction for symptomatology (−0.46), well-being (0.60), and social functioning (−0.43) and remained consistent across diagnoses.

**Discussion:**

The Brief INSPIRE-O demonstrated strong psychometric qualities and could be recommended as a measure of personal recovery for use in both research and clinical practice. Its strong theoretical basis and short completion time make it suitable for use for research. Incorporating Brief INSPIRE-O into clinical assessment will further support the process of mental health systems re-orientating towards personal recovery.

## 1 Introduction

The Comprehensive Mental Health Action Plan 2013–2030 by the World Health Organization (WHO) acts as a blueprint, aiding nations in implementing a person-centered, rights-based mental health strategy with an emphasis on personal recovery (1). Complementing this, the 2022 World Mental Health Report strongly advocates for further prioritizing personal recovery as the process of reclaiming a meaningful life against the backdrop of mental health challenges (2, 3). This can be achieved by empowering individuals to understand and control their own lives (4). Furthermore, the 2021 WHO Guidance on Community Mental Health Services offers insights and examples of aligning community-based services with international human rights standards, again promoting personal recovery (5). This agenda is aimed at empowering individuals living with mental health issues to renew hope and commitment, redefine identity, integrate illness into everyday life, promote involvement in activities, and provide social support and support for reintegration in the community (6–8).

Despite this, mental health services in many countries have predominantly concentrated on alleviating symptoms and managing mental health disorders. However, there is increasing consensus that personal recovery should be a key therapeutic objective and outcome within the mental health realm (9, 10). This evolving perspective indicates that individuals with mental health issues can realize substantial enhancements in their overall well-being, even if symptoms persist or still fit the diagnostic criteria for specific mental disorders (11–14). Notably, while the mental health discipline has started emphasizing personal recovery alongside traditional clinical objectives (15–18), there is still a significant need to further align routine clinical practices with the WHO’s recommendations (19). Therefore, it is recommended that service evaluation criteria incorporate indicators for the successful integration of personal recovery (20, 21).

Given the transition in the mental health landscape, it is important to have tools that can effectively measure personal recovery. However, a significant obstacle to advancing a recovery-focused approach in psychiatry is the scarcity of scientifically validated brief measures that can be easily implemented in busy clinical practice. The self-report measure Brief INSPIRE-O is specifically designed as an outcome scale to assess personal recovery within the context of mental health, where the “O” signifies its focus on outcomes.

Brief INSPIRE-O was adapted from the Brief INSPIRE (see details about the modifications in the *Methods* section), a patient-rated experience measure of staff support for personal recovery (22), based on the CHIME framework. CHIME identified five processes involved in recovery: Connectedness, Hope, Identity, Meaning, and Empowerment. The CHIME Framework was developed through a systematic review (10), and was then validated by current mental health service users (23) and cross-culturally (9). Brief INSPIRE and the more comprehensive INSPIRE measure are widely used widespread internationally and have been translated from the original English version into 27 languages (researchintorecovery.com/inspire).

This article presents a validation of the Brief INSPIRE-O, which is important for several reasons. First, it acknowledges the importance of personal recovery as a distinct and meaningful treatment target. Second, the findings may contribute to the advancement of personal recovery in mental health by providing clinicians, researchers, and policymakers with reliable and valid measurement tools for assessing personal recovery outcomes. Third, the development of a valid and reliable measure can facilitate the systematic collection of data on personal recovery from a variety of clinical settings, thereby contributing to expanding knowledge about personal recovery across a range of mental health problems.

This study evaluated the psychometric qualities of the Brief INSPIRE-O, including its reliability, scalability, and validity. A critical aspect of a measure’s psychometric qualities is test–retest reliability, investigating the measure’s stability and resistance to random measurement errors. Another aspect of psychometric validation is assessing the scalability of the measure. Mokken analysis is a probabilistic nonparametric approach that evaluates the scalability and hierarchical structure of items within a measure without making strict assumptions on the item response functions, as required by parametric item response theory methods, such as the Rasch model (24, 25). Using Mokken analysis, our study provides valuable insights into the Brief INSPIRE-O measure, highlighting the relative intensity of the items and their ability to discriminate between individuals along the recovery continuum. To investigate the degree to which the items of the Brief INSPIRE-O cohere, we analyzed their internal reliability. Finally, evaluating the construct validity of a scale involves determining how closely it aligns with other theoretically relevant constructs. The Brief-INSPIRE-O measure was expected to be positively associated with well-being, encapsulating an individual’s overall quality of life, as indexed by the World Health Organization Well-Being Index (WHO-5) (26). Furthermore, it was expected that Brief INSPIRE-O would have very small or negative associations with symptomatology (The Symptom Check List; SCL-10) and poor social functioning (Modified Shehan Disability Scale; SDS).

## 2 Methods

### 2.1 Study setting

The current paper utilized data from the Mental Health Services, Capital Region of Denmark (MHS-CR) which is the largest mental health service in Denmark, covering a catchment area of 1.85 million people with nine psychiatric treatment sites. In Denmark, psychiatric treatment in the secondary health sector is organized in treatment packages that specify relevant evidence-based treatments for specific diagnoses (27, 28). To monitor treatment effects, the MHS-CR developed an Internet-based monitoring system (IMS) that collects data pre- and post-treatment for all patients with non-psychotic disorders receiving treatment in a treatment package.

### 2.2 Participants and procedure

Initially, the patients underwent screening at a central visitation unit before being referred to the treatment site. At the treatment sites, psychiatrists and psychologists diagnose patients as part of routine clinical practice. All patients accepted for treatment for a range of mental health problems (including depression, anxiety disorders, and personality disorders) completed assessments using an Internet-based monitoring system (IMS) and were included in the study. Patients with psychotic disorders were not included in the monitoring system.

For the measurement of test–retest reliability, a subgroup of 61 participants was administered the Brief INSPIRE-O measure again 2–3 weeks after the first test. All data were recorded and stored according to the regional and national guidelines. The current study included all full pre-treatment datasets from the IMS between 1 March 2018 and 1 March 2020, leaving a dataset of 8,192 patients for inclusion in the study.

### 2.3 Materials

The Brief INSPIRE was modified to a patient-rated outcome measure called Brief INSPIRE-O. For each of the five items, the Brief INSPIRE item ‘My worker helps me with…’ was altered to leave out the reference to support from a professional. For example, ‘*My worker helps me feel supported by other people*’ was modified to ‘*I feel supported by other people.*’ Brief INSPIRE-O has the same scoring as Brief INSPIRE: rating is made on a 5-point Likert scale with (0) not at all, (1) not much, (2) somewhat, (3) quite a lot, and (4) very much, and the total scale scores are multiplied by 5 to range from 0 (low recovery) to 100. The 5-item Brief INSPIRE-O scale was translated into Danish, back-translated, and adapted to a PROM (29). In addition to Danish, it is available in Bosnian, Dutch, English, and Spanish languages. In a population study among Danish citizens, Moeller et al. (29) reported an internal consistency for Brief INSPIRE-O of 0.83 and a mean score of 71.1 with a standard deviation of 19.5. Moreover, Brief INSPIRE-O was positively correlated with the number of self-reported social contacts (*r* = 0.26) and self-reported general health (*r* = 0.54).

To measure the symptom burden, the SCL-10 (30) was used. The SCL-10 comprises five depression and five anxiety items from the SCL-90-R (31), which is a 90-item self-report symptom inventory that assesses psychological symptoms and distress. The total scale scores of the SCL-10 are multiplied by 2.5, ranging from 0 (low symptom load) to 100. The SCL-10 is a valid and reliable measure of symptom change in patients receiving treatment for depression or anxiety disorders (30).

For assessing subjective psychological well-being, WHO-5 was used. It is a self-report rating scale consisting of five positively phrased items scored on a Likert scale ranging 5 (all of the time) to 0 (none of the time). The total scale scores were multiplied by four to range from 0 (low well-being) to 100 (high well-being). The WHO-5 demonstrates good construct validity, and the scale was found to be a reliable indicator of subjective well-being across different settings and diagnoses and is sensitive in capturing improvements in well-being (32).

Measuring social functioning, the modified SDS (33), a three-item, self-report global measure assessing work/studies, social life, and family life, was used. Disruption due to symptom burden was rated from 0 (not at all) to 10 (extremely) on each of the areas of functioning, resulting in a total scale score ranging from 0 (low disability) to 30 (severe disability). The scale has demonstrated strong psychometric qualities, including its ability to discriminate between active and inactive treatments (34).

### 2.4 Data analysis

The study population was described in terms of sex, age, and primary diagnosis. To analyze the test–retest reliability of the Brief-INSPIRE-O, a mixed effects model of the Brief-INSPIRE-O scale against time with repeated measurements of the same patient was performed. The intraclass correlation coefficient (ICC) with 95% confidence interval was calculated from the variance components of the model. ICC values greater than 0.90 are regarded as excellent reliability, values between 0.75 and 0.90 as good reliability, and values between 0.50 and 0.75 as moderate reliability.

A non-parametric Mokken analysis of the Brief INSPIRE-O was conducted to investigate the properties of the individual items according to the total scale (25) For each item, trace lines were visually inspected, and the monotonicity criterion was calculated to assess the assumption of monotonicity. A maximum value below 40 across all items of the monotonicity criterion is regarded as acceptable. Further, we examined whether the item difficulty was invariant for all values of the total scale by inspecting the estimated probabilities of ordering all pairs of the five Brief INSPIRE-O items for all values of the total scale. Finally, the scalability of the Brief INSPIRE-O items and the total scale was measured using Loevinger’s coefficient of homogeneity (*H*), which is defined as *H* = 1- “number of observed Guttman’s errors”/”number of expected Guttman’s errors” (35, 36). A Guttman error occurs when a participant scores less on an item with low difficulty than on one with high difficulty. Values of *H* at 0.40 or higher are regarded as clear indication of scalability, values between 0.30 and 0.39 as acceptable, and values between 0.20 and 0.29 as questionable. The doubly monotonicity homogeneous model of Brief INSPIRE-O may be assumed if the monotonicity assumption is satisfied, the order of item difficulty is invariant for all values of the total scale, and scalability is acceptable.

Summary statistics (mean, standard error, median, and interquartile range) were calculated for the Brief INSPIRE-O and the mental health scales SCL-10, WHO-5, and SDS. The internal consistency of each scale was assessed using Cronbach’s alpha (37) and McDonald’s omega [based on confirmatory factor analysis and estimated using maximum likelihood (38)]. Values of internal consistency above 0.9 are regarded as excellent, values between 0.80 and 0.90 as good, values between 0.70 and 0.80 acceptable, values between 0.60 and 0.70 as questionable, values between 0.50 and 0.60 as poor, and values below 0.50 as unacceptable.

Finally, to examine convergent and divergent validity across treatment diagnoses, Pearson’s correlations between the Brief INSPIRE-O and the mental health scales SCL-10, WHO-5, and SDS were calculated for the total sample and separately for each diagnosis.

All analyses were performed using Stata version 18.0. A two-sided significance level of 5% was used.

## 3 Results

The clinical characteristics of the study participants are presented in **Table 1**. More than three-quarters of the patients were females, and the most prevalent diagnoses were depression (33.7%) and anxiety (27.6%).

**Table 1:** Participant characteristics at baseline (n = 8,192).

Characteristic	Patients
Total, n	8.192
Sex, n (%)	
*Male*	1.903 (23.2)
*Female*	6.289 (76.8)
Age, mean (SD)	32.9 (11.7)
Primary diagnosis, n (%)	
*Depression*	2.556 (33.7)
*Anxiety*	2.094 (27.6)
*Personality disorder*	1.484 (19.6)
*PTSD*	725 (9.6)
*Eating disorder*	584 (7.7)
*Other*	142 (1.9)

Missing (n): diagnosis (607).

### 3.1 Test–retest reliability

The test–retest reliability of Brief Inspire-O was evaluated on a subsample of the study population (n = 61); the reliabilities of the total scale as well as all single items were moderate, ranging between 0.50 and 0.75 (see **Table 2**).

**Table 2:** Test–retest reliability of Brief INSPIRE-O (n = 61).

	Test–retest ICC (95% CI)
Brief Inspire-O total scale	0.75 (0.63, 0.85)
Brief Inspire-O single items	
Item 1: I feel supported by other people	0.51 (0.33, 0.69)
Item 2: I have hopes and dreams for the future	0.71 (0.58, 0.82)
Item 3: I feel good about myself	0.74 (0.61, 0.84)
Item 4: I do things that mean something to me	0.50 (0.32, 0.68)
Item 5: I feel in control of my life	0.54 (0.37, 0.71)

ICC, Intraclass correlation; CI, confidence interval.

The highest test–retest reliability ICCs were for item 2 (“I have hopes and dreams for the future”; 0.71) and item 3 (“I feel good about myself”; 0.74), while the other three items ranged from 0.50 to 0.54 (see **Table 2**).

### 3.2 Scalability

Visual inspection of item trace lines (not shown) and a maximum monotonicity criterion of 22 (Item 1), well below the threshold of 40, indicated that the monotonicity assumption was satisfied. Additionally, the assumption of invariance of item difficulty for all values of the Brief-INSPIRE-O was satisfied, with no violations of the assumption (not shown). Finally, the scalability assumption was satisfied with scale-*H* = 0.39 > 0.30, and no item-*H* < 0.30.

The highest scalability scores were for item 3 (“I feel good about myself;” *H =* 0.429) and item 4 (“I do things that mean something to me;” *H =* 0.431), while the lowest was for item 1 (“I feel supported by other people;” *H =* 0.302). The highest mean score was for item 1 (“I feel supported by other people,” mean *=* 2.32, difficulty = 0.045), and the lowest was for item 5 (“I feel in control of my life”; mean *=* 0.93, difficulty = 0.389). The order of difficulty for the Brief-INSPIRE-O measure was from the most difficult to the least: Items 5, 3, 2, 4, and 1 (see **Table 3**).

**Table 3:** Mokken analysis of Brief-INSPIRE-O (n = 8,192).

			Guttman errors		Loevinger’s *H* coefficient	
Item^a^	Mean (SD)	Difficulty	Observed	Expected		*p*-value
1	2.32 (1.08)	0.0452	21,953	31,473.54	0.30249	<0.001
2	2.06 (1.21)	0.0989	21,042	35,043.88	0.39955	<0.001
3	1.04 (0.93)	0.3359	16,193	28,337.09	0.42856	<0.001
4	1.62 (0.94)	0.0953	16,195	28,440.00	0.43056	<0.001
5	0.93 (0.91)	0.3887	16,441	27,637.93	0.40513	<0.001
Total scale			45,912	75,466.22	0.39162	<0.001

a Item range 0–4.

### 3.3 Internal consistency and descriptive statistics of all scales

Internal consistency for the measures were all acceptable with Cronbach’s alpha ranging between 0.73 (Brief INSPIRE-O) and 0.81 (WHO-5) (see **Table 4**).

**Table 4:** Summary statistics and internal consistency of all scales at baseline.

		Summary statistics		Internal consistency	
Scale	Range	Median (IQR)	Mean (SD)	Cronbach’s alpha	McDonald’s omega
Brief Inspire-O, n = 8,192	0–100	40 (25)	39.9 (17.6)	0.73	0.74
SCL-10, n = 8,169	0–100	57.5 (22.5)	57.8 (17.0)	0.78	0.79
SDS, n = 8,157	0–30	21 (8)	20.3 (6.1)	0.81	0.82
WHO-5, n = 8,184	0–100	24 (20)	27.1 (16.7)	0.81	0.81

### 3.4 Convergent and divergent validity

All correlations were stable across treatment diagnoses (**Table 5**). The results of the correlations with the Brief Inspire-O across different diagnoses showed a stronger negative correlation with the SCL-10 than expected ranging from −0.410 for personality disorder to −0.516 for PTSD, and for the modified SDS ranging from −0.374 for personality disorder to −0.436 for anxiety. The expected positive correlation between Brief Inspire-O and WHO-5 was strongest for personality disorder (0.567) and weakest for depression (0.546).

**Table 5:** Convergent and divergent validity of Brief INSPIRE-O stratified by diagnosis.

		Diagnosis				
	Total, n = 8,192	Anxiety, n = 2,082	Depression, n = 2,552	Personality disorder, n = 1,469	Eating disorder, n = 583	PTSD, n = 734
Correlations with Inspire total scale						
*SCL-10*	−0.457	−0.487	−0.438	−0.410	−0.439	−0.516
*SDS*	−0.428	−0.436	−0.382	−0.374	−0.423	−0.419
*WHO-5*	0.604	0.600	0.546	0.567	0.609	0.585

All correlations are highly statistically significant (all p < 0.001).

## 4 Discussion

In this study, Brief INSPIRE-O displayed strong psychometric qualities. Good scalability and satisfactory internal consistency indicate that it is reliable. Its moderate test–retest reliability is comparable to that of other measures of personal recovery (39). The higher test-retest ICCs for Items 2 (“I have hopes and dreams for the future”) and 3 (“I feel good about myself”) compared to the other three items indicate higher stability, suggesting that these two items may be relatively more resistant to change. The pattern seen for Item 1 (“I feel supported by other people “) exhibit the highest mean score and lowest difficulty, yet a relatively low test–retest reliability and the lowest scalability score, could suggest a distinctiveness from the other items, which is further underscored by it being the only item that incorporates the mention of other people in its wording. Interestingly, the items ranked as the most difficult pertained to one’s sense of agency and self-worth. Specifically, Item 5 (“I feel in control of my life “) and Item 3 (“I feel good about myself “) indicate that feelings of autonomy and positive self-regard are the most challenging to attain for patients with non-psychotic disorders. Notably, the correlations between Brief INSPIRE-O and other recognized mental health criteria validated its role in assessing personal recovery, including evidence of both convergent and divergent validity. The consistency of correlations across treatment diagnoses underscores the robustness of the Brief INSPIRE-O measure. However, the strong negative correlation between SCL-10 and PTSD might suggest a weaker association between personal recovery and distress in PTSD. Similarly, the pronounced negative relationship with the modified SDS for anxiety could indicate a lesser link between personal recovery and function in anxiety compared to other diagnoses. Conversely, the strongest positive correlation with the WHO-5 for personality disorders and lowest for depression could suggest that personal recovery and well-being align best among personality disorders and least among patients with depression. Consistent with the understanding that personal recovery is often diminished in persons with mental health issues, the Brief INSPIRE-O’s average score was notably lower in this treatment-seeking population than in the general population’s mean of 71 (29).

### 4.1 Implications for modern psychiatric care

Given its strong psychometric qualities, the Brief INSPIRE-O can be adopted in both clinical and research settings. By implementing this measure in clinical trials or evaluations, researchers can gain insight into how interventions influence personal recovery, especially when used with complementary qualitative methodologies such as diary studies (40). This knowledge can guide clinicians towards designing treatment plans that prioritize holistic, person-centered care, ensuring a comprehensive approach to mental well-being. Its use in clinical practice could potentially improve the assessment and monitoring of mental health conditions with treatment planning and patient care oriented towards personal recovery (41). At an individual level, a clinician can collaborate with a patient to identify the areas of the CHIME framework that are most significant to that particular patient. Through open dialogue and active listening, clinicians can understand the patient’s unique needs and preferences. Together, they can prioritize these aspects and develop a tailored intervention plan that specifically targets the areas of the CHIME framework that are most important to the patient’s personal recovery journey. This collaborative approach ensures that the treatment plan aligns with the patient’s goals and promotes a sense of empowerment and ownership during the recovery process.

Finally, the Brief INSPIRE-O is brief enough to be administered for continuous outcome measurement (42) and has the potential to be included in internationally agreed standards for quality and outcome monitoring (43).

### 4.2 Strengths and limitations

A strength of this study is the large and diverse sample, offering comprehensive insight into the performance of the Brief INSPIRE-O in various mental health disorders. However, the psychometric adequacy of the Brief INSPIRE-O when used in patients with psychosis remains to be evaluated. Future research should aim to further explore the qualities of the Brief INSPIRE-O in different populations and settings, and extend the test–retest analysis to a larger sample. Studies using a longitudinal design to measure changes over time are crucial. Finally, because the importance of personal recovery is a global agenda, it would also be helpful to conduct an international validation study on Brief INSPIRE-O, considering its availability in various language versions.

## 5 Conclusions

In summary, the Brief INSPIRE-O exhibits strong psychometric qualities, making it a reliable tool for assessing personal recovery in mental health care across a range of clinical disorders. Its scalability, internal consistency, and moderate test–retest reliability contribute to its credibility. While certain items demonstrated higher stability and others revealed distinct patterns, correlations with established mental health criteria validated their role in assessing personal recovery.

A wider implementation of this measure may have significant implications for modern psychiatric care, enabling informed clinical decisions and guiding research. The brief administration time facilitates continuous outcome measurement and potential inclusion in international standards for quality and outcome monitoring. However, further international evaluation in diverse populations and settings are necessary to confirm its utility and reliability.

## Data availability statement

The raw data supporting the conclusions of this article will be made available by the authors, without undue reservation.

## Ethics statement

The studies involving humans were approved the institutional review board of the Mental Health Service, Capital Region, Denmark. The studies were conducted in accordance with the local legislation and institutional requirements. Written informed consent for participation was not required from the participants or the participants’ legal guardians/next of kin because it was a retrospective study without contact to participating patients.

## Author contributions

SM: Conceptualization, Funding acquisition, Methodology, Project administration, Writing – original draft. PL: Formal analysis, Methodology, Writing – review & editing. SA: Conceptualization, Writing – review & editing. MS: Writing – review & editing. IA: Writing – review & editing. MA: Formal analysis, Writing – review & editing. SS: Conceptualization, Funding acquisition, Methodology, Project administration, Writing – review & editing.
